# Supplementary material for: Using social capital to address youth sexual and reproductive health and rights in disaster preparedness and response: A qualitative study highlighting the strengths of Pacific community organisations and networks
Source: PLOS Glob Public Health. 2023 May 5;3(5):e0001624. doi: 10.1371/journal.pgph.0001624 (PMC10162552; doi:10.1371/journal.pgph.0001624)
Supplement: S1 Appendix — (DOCX) [file pgph.0001624.s001.docx]

**Interview guide for service provider qualitative interviews**

**Interviewer to read**

*Thank you for participating in this research. I’m interested in hearing about your perspectives as you were involved in the TC Harold responses in the Pacific. Specifically I’m interested in your experiences and thoughts about youth and their sexual and reproductive health and rights (SRHR) and youth engagement during disasters such as TC Harold. I would like to know whether youth SRHR is an issue of concern, and what might help strengthen future responses to make sure youth are supported to protect their health and bodies.*

*When referring to youth I’m using the age group 15-24 years, and SRHR includes all areas related to the ability of youth to protect themselves from harm and seek information, health services and support related to their bodies and their sexual and reproductive health. So this includes things like sexual and gender based violence (SGBV), sexually transmitted infections (STIs), complications related to pregnancy and birth, early marriage, menstrual hygiene management, gender inequality and sexual exploitation and abuse. I’m interested in the experiences of all youth, including young women, young men, and other marginalised groups such as those living with disabilities, and those with gender and sexual diversity. When I talk about youth engagement I’m interested in exploring inclusive, intentional, mutually respectful partnerships with youth.*

*Before we begin the interview questions I will just ask a few general questions about you and your role within the organisation. This will not appear in the interview transcript.*

| Type of interview (e.g. phone/skype/WhatsApp) | Duration of interview | Age | Gender | Type of organisation (eg INGO, CSO, government) | Role in organisation | Years in role |
| --- | --- | --- | --- | --- | --- | --- |
|  |  |  |  |  |  |  |
|  |  |  |  |  |  |  |

General questions

Now I’m going to ask a few quick questions about your organisation and the work that you did during TC Harold. You don’t have to tell me any names or identifying information, but just the types of services you delivered and in which countries and provinces.

- Can you tell me about your organisation and the type of work that you do? Are you an international, national, government, non-government organisation, or community network?
  - Tell me about you/your organisations response to TC Harold. How were you involved?
  - Which sectors or issues did you work on during the TC Harold response?
  - Were there any specific issues that you focused on and why?
  - Did you think anything could have been improved in the response related to (the issues they work on)
    - If yes, what and why?
    - If no, why do you think it worked well?

SRHR needs during TC Harold responses

These next few questions will focus on SRHR needs in general that you may have noticed or responded to during TC Harold.

- Can you tell me about any SRHR related needs that you observed or responded to during TC Harold?
  - Probe about experiences in responding to any issues related to protection, gender, safety and health
- What were the most concerning SRHR needs you noticed?
  - *Prompts: SGBV, early marriage, sexual exploitation*
- Which groups of people were mostly affected by these issues?
  - *Prompts: Women and girls, rural and remote communities, diverse SOGIEs, people with disability*
- What were the main challenges in the response to (issues identified)?
  - What were the challenges?
  - Why were they challenges?
  - What could have been done better for future responses?
  - What about some of the things that went well? Why did they go well?

*Prompts:*

- - *Knowing where to refer to, sociocultural barriers or stigma, confidentiality, collaboration between sectors and organisations, legal concerns*

Youth SRHR

These next few questions explore your experiences and views on youth and SRHR during TC Harold responses.

- In your opinion, how easy or difficult is it to identify SRHR issues specific to youth?
  - Why do you think it’s easy/hard?
  - Can you give me an example of something that was easy?
  - Can you give me an example of something that was hard?

*Prompts:*

- - *Were certain groups prioritised or considered according to age, sex or gender during responses?*
  - *Was youth SRHR a priority or an issue of consideration during responses?*
- In your opinion, how important is it to address youth SRHR during disaster responses?
  - Why is it important/not important?
- What are the main barriers or challenges to addressing youth SRHR issues during disaster responses?

*Prompts:*

- - *Age, society, religion or culture, policy or legal issues, coordination and collaboration between sectors and organisations, not knowing who to refer to*

Youth engagement

Now we will discuss youth engagement in TC Harold responses.

- Can you tell me about any experiences where you or your organisations worked with youth staff members or volunteers?
- What types of activities were youth involved in during TC Harold?

*Prompts:*

- - *Delivering services, information and communication, assessment teams*
- Can you tell me about some of the difficulties in meaningfully engaging with youth during disaster responses?

*Prompts:*

- - *Are youth encouraged to participate or are they excluded?*
- In your opinion, how important is it to involve youth in identifying and addressing youth SRHR issues for themselves and their peers during disaster responses?
  - Why is it important/not important?

Challenges and opportunities to strengthening responses to youth SRHR

Finally, these next few questions will focus on your views about where and how interventions can be made to strengthen responses to youth SRHR in future disasters.

- In your opinion, what are the main challenges to identifying and reducing youth-specific SRHR risks and negative outcomes following disasters?
- Thinking about your experiences during TC Harold responses, what are your suggestions for possible opportunities to strengthen responses to youth SRHR issues?
- What would you like to see happen in future responses in order to improve identification and service provision for youth in relation to their SRHR?

*That brings us to the end of the interview. Before we finish, is there anything else you would like to talk about or add? Any other ideas or comments about anything we have discussed?*

*Thank you very much for your time today. I really appreciate you taking part in this research. Would you like me to present the findings to your team once we have finished this research?*
