# Supplementary material for: Using social capital to address youth sexual and reproductive health and rights in disaster preparedness and response: A qualitative study highlighting the strengths of Pacific community organisations and networks
Source: PLOS Glob Public Health. 2023 May 5;3(5):e0001624. doi: 10.1371/journal.pgph.0001624 (PMC10162552; doi:10.1371/journal.pgph.0001624)
Supplement: Attachment — Submitted filename: Response to Reviewers.docx [file pgph.0001624.s002.docx]

Dear Editors and Reviewers,

Many thanks for the constructive feedback on our paper. We have addressed the point-by-point feedback below and revised the manuscript accordingly. We appreciate the feedback and believe that it has strengthened our paper.

Sincerely,

Nabreesa Murphy, on behalf of the authors

| Editor’s feedback | Author’s response |
| --- | --- |
| 1. Please review reference list to ensure that it is complete and correct. If you have cited papers that have been retracted, please include the rationale for doing so in the manuscript text, or remove these references and replace them with relevant current references. Any changes to the reference list should be mentioned in the rebuttal letter that accompanies your revised manuscript. If you need to cite a retracted article, indicate the article’s retracted status in the References list and also include a citation and full reference for the retraction notice. | Thank you. We have added the DOI to reference 25 which was previously under review and has now been accepted. We hope the rest of the reference list is acceptable. |
| 2. Please amend your detailed Financial Disclosure statement. This is published with the article. It must therefore be completed in full sentences and contain the exact wording you wish to be published.   a. State the initials, alongside each funding source, of each author to receive each grant. b. State what role the funders took in the study. If the funders had no role in your study, please state: “The funders had no role in study design, data collection and analysis, decision to publish, or preparation of the manuscript.” c. If any authors received a salary from any of your funders, please state which authors and which funders.  If you did not receive any funding for this study, please simply state: “The authors received no specific funding for this work.” | Thank you. We have amended the Financial Disclosure statement as follows:  *This work was supported by the University of Melbourne (Human Rights Scholarship to NM, Population Health Investing in Research Student's Training [PHIRST] to NM, Dame Kate Campbell Fellowship to MAB), and the Australian Research Council (Discovery Early Career Researcher Award [DE200100264] to MAB). The funders had no role in study design, data collection and analysis, decision to publish, or preparation of the manuscript.* |
| 3. In the Funding Information you indicated that no funding was received. Please revise the Funding Information field to reflect funding received. | Thank you. We have amended as stated above. |
| 4. We have noticed that you have uploaded Supporting Information files, but you have not included a list of legends. Please add a full list of legends for your Supporting Information files after the references list. | Thank you. We have added the following under Reference list:  ***Supporting Information files***  *S1 Appendix A – Interview guide S2 Figure 1 – How community organisations and networks utilised strengths in social capital to mitigate challenges to youth SRHR.* |
| Reviewer’s feedback | Author’s response |
| Q1. Does this manuscript meet PLOS Global Public Health’s publication criteria? Is the manuscript technically sound, and do the data support the conclusions? The manuscript must describe methodologically and ethically rigorous research with conclusions that are appropriately drawn based on the data presented. | |
| Reviewer 1: Yes | Thank you. No revisions made. |
| Reviewer 2: Yes | Thank you. No revisions made. |
| Q2. Has the statistical analysis been performed appropriately and rigorously? | |
| Reviewer 1: N/A | No revisions made. |
| Reviewer 2: N/A | No revisions made. |
| Q3. Have the authors made all data underlying the findings in their manuscript fully available (please refer to the Data Availability Statement at the start of the manuscript PDF file)?  The [PLOS Data policy](https://journals.plos.org/globalpublichealth/s/data-availability) requires authors to make all data underlying the findings described in their manuscript fully available without restriction, with rare exception. The data should be provided as part of the manuscript or its supporting information, or deposited to a public repository. For example, in addition to summary statistics, the data points behind means, medians and variance measures should be available. If there are restrictions on publicly sharing data—e.g. participant privacy or use of data from a third party—those must be specified. | |
| Reviewer 1: No | Thank you. We have added the following to the Data Availability Statement:  *The data/transcripts are not publicly available due to the need to protect participant confidentiality. De-identified excerpts from transcripts may be made available on reasonable request. Requests can be directed to the University of Melbourne Centre for Health Equity (*[*che-enquiry@unimelb.edu.au*](mailto:che-enquiry@unimelb.edu.au)*).* |
| Reviewer 2: Yes | As above |
| Q4. Is the manuscript presented in an intelligible fashion and written in standard English?  PLOS Global Public Health does not copyedit accepted manuscripts, so the language in submitted articles must be clear, correct, and unambiguous. Any typographical or grammatical errors should be corrected at revision, so please note any specific errors here. | |
| Reviewer 1: Yes | Thank you. No revisions made. |
| Reviewer 2: Yes | Thank you. No revisions made. |
| Q5. Review Comments to the Author  Please use the space provided to explain your answers to the questions above. You may also include additional comments for the author, including concerns about dual publication, research ethics, or publication ethics. (Please upload your review as an attachment if it exceeds 20,000 characters) | |
| Reviewer 1: Authors should make excerpts of the transcripts relevant to the study available in an appropriate data repository, within the paper, or upon request if they cannot be shared publicly. If even sharing excerpts would violate the agreement to which the participants consented, authors should explain this restriction and what data they are able to share in their Data Availability Statement.  There should be some form of statement about the excertps of the transcripts. Options are listed below:  The datasets generated during and/or analysed during the current study are available in the [NAME] repository, [PERSISTENT WEB LINK TO DATASETS].  The datasets generated during and/or analysed during the current study are available from the corresponding author on reasonable request.  All data generated or analysed during this study are included in this published article (and its supplementary information files).  The datasets generated during and/or analysed during the current study are not publicly available due to [REASON(S) WHY DATA ARE NOT PUBLIC] but are available from the corresponding author on reasonable request. | Thank you for your feedback. We have added the following to the Data Availability Statement:  *The data/transcripts are not publicly available due to the need to protect participant confidentiality. De-identified excerpts from transcripts may be made available on reasonable request. Requests can be directed to the University of Melbourne Centre for Health Equity (*[*che-enquiry@unimelb.edu.au*](mailto:che-enquiry@unimelb.edu.au)*).* |
| Reviewer 2: This manuscript fills a gap in research associated with sexual and reproductive health needs and rights of adolescents in areas prone to natural disasters. Statistical analyses were not needed, as this qualitative data collection used appropriate qualitative data analyses to present their results. This research will serve as the basis for additional quantitative and quantitative research documenting SRHR among adolescents in fragile conditions, which should serve as the basis for building programs to mitigate these adolescents' challenges. This research clearly shows that partnering with local NGOs already working with adolescents will improve and strengthen approaches by international disaster relief organizations when they come in during disaster relief operations. This is a well-written manuscript which needs minor editing for a couple of typos (i.e.: P11, line 257; says "cased" which should be "cases". This research could have been strengthened by additional interview with people from Tonga--but the authors rightfully point out that this is a limitation. | Thank you. We have addressed the typing errors. |
