# Supplementary material for: Using social capital to address youth sexual and reproductive health and rights in disaster preparedness and response: A qualitative study highlighting the strengths of Pacific community organisations and networks
Source: PLOS Glob Public Health. 2023 May 5;3(5):e0001624. doi: 10.1371/journal.pgph.0001624 (PMC10162552; doi:10.1371/journal.pgph.0001624)
Supplement: Attachment — Submitted filename: Response to Reviewers.docx [file pgph.0001624.s003.docx]

Dear Editors and Reviewers,

Many thanks for the constructive feedback on our paper. We have addressed the point-by-point feedback below and revised the manuscript accordingly. We appreciate the feedback and believe that it has strengthened our paper.

Sincerely,

Nabreesa Murphy, on behalf of the authors

| Journal requirements | Author’s response |
| --- | --- |
| 1. Please review reference list to ensure that it is complete and correct. If you have cited papers that have been retracted, please include the rationale for doing so in the manuscript text, or remove these references and replace them with relevant current references. Any changes to the reference list should be mentioned in the rebuttal letter that accompanies your revised manuscript. If you need to cite a retracted article, indicate the article’s retracted status in the References list and also include a citation and full reference for the retraction notice. | Thank you. We have added the full reference details to reference 25 which has now been accepted and published. We hope the rest of the reference list is acceptable. |
| Reviewer’s feedback | Author’s response |
| Q1. If the authors have adequately addressed your comments raised in a previous round of review and you feel that this manuscript is now acceptable for publication, you may indicate that here to bypass the “Comments to the Author” section, enter your conflict of interest statement in the “Confidential to Editor” section, and submit your "Accept" recommendation. | |
| Reviewer 1: All comments have been addressed. | Thank you. No revisions made. |
| Q2. Does this manuscript meet PLOS Global Public Health’s publication criteria? Is the manuscript technically sound, and do the data support the conclusions? The manuscript must describe methodologically and ethically rigorous research with conclusions that are appropriately drawn based on the data presented. | |
| Reviewer 1: Yes | Thank you. No revisions made. |
| Q3. Has the statistical analysis been performed appropriately and rigorously? | |
| Reviewer 1: N/A | No revisions made. |
| Q4. Have the authors made all data underlying the findings in their manuscript fully available (please refer to the Data Availability Statement at the start of the manuscript PDF file)?  The [PLOS Data policy](https://journals.plos.org/globalpublichealth/s/data-availability) requires authors to make all data underlying the findings described in their manuscript fully available without restriction, with rare exception. The data should be provided as part of the manuscript or its supporting information, or deposited to a public repository. For example, in addition to summary statistics, the data points behind means, medians and variance measures should be available. If there are restrictions on publicly sharing data—e.g. participant privacy or use of data from a third party—those must be specified. | |
| Reviewer 1: Yes | Thank you. No revisions made. |
| Q5. Is the manuscript presented in an intelligible fashion and written in standard English?  PLOS Global Public Health does not copyedit accepted manuscripts, so the language in submitted articles must be clear, correct, and unambiguous. Any typographical or grammatical errors should be corrected at revision, so please note any specific errors here. | |
| Reviewer 1: Yes | Thank you. No revisions made. |
| Q6. Review Comments to the Author  Please use the space provided to explain your answers to the questions above. You may also include additional comments for the author, including concerns about dual publication, research ethics, or publication ethics. (Please upload your review as an attachment if it exceeds 20,000 characters) | |
| Reviewer 1: Thank you for your well written, analyzed, and discussed research manuscript. I have some minor points that need to be revised.  Line 4-7 – please create two sentences out of the one sentence to break up your ideas.  Line 34- “increase risks” should be more specific. What are the increased risks?  Line 66 – Can you be more specific regarding the multi-level factors that challenge youth SRHR. It’s a broad statement and the sentences that follow don’t link to the multi-level factors.    Line 182 – you say all interviews were audio recorded and transcribed. However, earlier you say one interview was via email communication. Can you change the wording of this sentence as it was not all interviews.    Line 277 and several others – you say several times that participants described how social capital …… I am assuming that participants did not use the words social capital but in your analysis due to your framework you analyzed it to be social capital. So a change of wording in necessary in these sections were you say participants described. Something like participants described what we determined to be social capital.  Line 322-323 – This entire section how does it relate to climate change disasters. It seems very general and not related. Can you make some connections in this section.  Line 339 – “community members supported and enabled safe access to water, sanitation, and hygiene (WASH) facilities.” How? Be more specific with your examples. It will help to give the reader more context.  Line 445 - Trained them in what? Do you have more details/context. | Thank you for your feedback. We have addressed each point below and have also amended the manuscript accordingly.  Thank you. We have made the sentence more succinct to reflect the suggested point (lines 4-6 in Revised Manuscript) and amended it as below:  *“Climate impacts are inequitable, with countries and communities who contribute the least to the climate crisis disproportionately facing increasing disaster risks that threaten their health and human rights.”*  Thank you. We have removed “increase risks” and amended the sentence to clarify the suggested point. The amended sentence (lines 35 – 37 in Revised Manuscript) now states:  *“Menstrual health taboos, and violence and discrimination against people with diverse sexual orientation, gender identity and expression (SOGIEs), increase challenges to fulfilling youth SRHR.”*  Thank you. We have amended the sentence (lines 68-71 in Revised Manuscript) to reflect the suggested point which now reads:  *“These models provide information and increase SRHR knowledge, address multi-level factors related to unequal power dynamics, sociocultural norms, and government policies that challenge youth SRHR, and facilitate access to clinical services.”*  Thank you. We have clarified further (lines 186 – 189 in Revised Manuscript) to reflect the suggested point, as below:  *“All interviews conducted over Zoom or telephone were audio-recorded and transcribed verbatim by NM, aided by an automated transcription service (Otter.ai). The e-mail interview followed the same question and answer format, which we directly transferred to a Word document and did not require further transcription.”*  Thank you. We have amended the wording throughout to clarify that ‘social capital’ as a phrase was not used by participants. Noting that qualitative reporting models combine ‘results’ and ‘discussion’ to provide a cohesive analytic narrative throughout, we hope the amendments sufficiently reflect the suggested point. As an example, the sentence (lines 283 – 285 in Revised Manuscript) now reads as below:  *“Participant accounts demonstrated how social capital in the form of youth volunteers and networks helped address challenges related to geographical remoteness, difficulty accessing funds on time, and time restrictions related to humanitarian response funds.”*  Thank you. This section intends to illustrate how existing challenges related to the taboo nature of youth SRHR increases challenges in disaster contexts. We have amended the sentence (lines 330 – 331 in Revised Manuscript) to reflect the suggested point, which now states:  *“All participants identified SRHR and youth SRHR issues as taboo subjects in many of their contexts, highlighting how existing challenges to realisation of SRHR are exacerbated in disasters.”*  We have also amended the last sentence of the same paragraph (lines 334 – 336) to make the connection to disasters, as below:  *“Josefa discussed navigated these challenges by being strategic about how they approached and involved village headmen and community leaders when addressing SRHR issues after disasters.”*  Thank you. We have clarified and expanded the sentence to two sentences (lines 350 – 355 in Revised Manuscript), as below:  *“For instance, community members accompanied and protected each other to facilitate safe access to water, sanitation, and hygiene (WASH) facilities, especially if they were located outside. Participants described community efforts to ensure no one had to travel to WASH facilities alone or in the dark, to minimise risks of violence for women, people with disabilities, and those with diverse SOGIEs.”*  Thank you. We have added a sentence before the participant quote to reflect the suggested point. The sentence (lines 464 – 466 in Revised Manuscript), provides more context as below:  *“She described how her organisation builds the capacity of youth to engage with community leaders, raising awareness about SRHR issues and focusing on addressing root causes of sexual and gender-based violence.”* |
